# Development and validation of a survival nomogram for patients with Siewert type II/III adenocarcinoma of the esophagogastric junction based on real-world data

**DOI:** 10.1186/s12885-021-08249-x

**Published:** 2021-05-10

**Authors:** Jian Chen, Yu-Jian Xia, Tian-Yu Liu, Yuan-Hui Lai, Ji-Shang Yu, Tian-Hao Zhang, Shiyin Ooi, Yu-Long He

**Affiliations:** 1 Center for Gastrointestinal Surgery, the First Affiliated Hospital of Sun Yat-Sen University, 58 Zhongshan 2nd Road, Guangzhou, 510080 Guangdong China; 2 Department of Obstetrics and Gynecology, the First Affiliated Hospital of Sun Yat-sen University, Guangzhou, Guangdong China; 3 Department of Thyroid and Breast Surgery, the Eastern Division of the First Affiliated Hospital of Sun Yat-sen University, Guangzhou, Guangdong China; 4 Digestive Medicine Center, the Seventh Affiliated Hospital of Sun Yat-Sen University, Shenzhen, Guangdong China

**Keywords:** Adenocarcinoma of the esophagogastric junction, Nomogram, Prognosis, Clinical decision support, Real-world data

## Abstract

**Background:**

The clinical staging systems for adenocarcinoma of the esophagogastric junction (AEG) are controversial. We aimed to propose a prognostic nomogram based on real-world data for predicting survival of Siewert type II/III AEG patients after surgery.

**Methods:**

A total of 396 patients with Siewert type II/III AEG diagnosed and treated at the Center for Gastrointestinal Surgery, the First Affiliated Hospital, Sun Yat-sen University, from June 2009 to June 2017 were enrolled. The original data of 29 variables were exported from the electronic medical records system. The nomogram was established based on multivariate Cox regression coefficients, and its performance was measured using Harrell’s concordance index (C-index), receiver operating characteristic (ROC) curve analysis and calibration curve.

**Results:**

A nomogram was constructed based on nine variables. The C-index for overall survival (OS) prediction was 0.76 (95% CI, 0.72 to 0.80) in the training cohort, in the validation-1 cohort was 0.79 (95% CI, 0.72 to 0.86), and 0.73 (95% CI, 0.67 to 0.80) in the validation-2 cohort. Time-dependent ROC curves and calibration curves in all three cohorts showed good prognostic predictive accuracy. We further proved the superiority of the nomogram in predictive accuracy for OS to pathological TNM (pTNM) staging system and other independent prognostic factors. Kaplan-Meier survival curves demonstrated the pTNM stage, grade of differentiation, positive lymph node, log odds of positive lymph node and organ invasion were prognostic factors with good discriminative ability.

**Conclusion:**

The established nomogram demonstrated a more precise prognostic prediction for patients with Siewert type II/III AEG.

**Supplementary Information:**

The online version contains supplementary material available at 10.1186/s12885-021-08249-x.

## Background

In recent decades, an increasing trend has been observed in the prevalence of adenocarcinoma of the esophagogastric junction (AEG), with a greater metastatic probability and worse survival than most cancers [1, 2]. AEG was classified by Siewert et al. [3] into three categories according to the position of the tumor epicenter with respect to the esophagogastric junction (EGJ). Nevertheless, the approach to the management of AEG has yet to be standardized. The 8th Union for International Cancer Control (UICC) and American Joint Committee on Cancer (AJCC) tumor-node-metastasis (TNM) classification recommended that AEG tumors whose epicenter was in proximal 2 cm of the cardia were staged as esophageal, while tumors whose epicenter was greater than 2 cm distal from the EGJ were staged using the gastric cancer TNM staging, even with the involvement of the EGJ [4, 5]. However, given the specific pathological features of AEG, oncologic surgical principles that are developed for esophageal or gastric cancer should not be simply applied to AEG. Therefore, it is of great significance to construct its clinical staging system for decision-making and prognostication.

Currently, the prognostic value of nomograms has been identified in various cancer types [6–8]. Adopting nomograms for predicting prognosis and clinical decision-making has compared favorably to the classical staging systems in different cancers [9, 10]. Hence, it has been widely proposed in clinical application as a substitute or even as a new criterion. Zhou et al. [11] developed a nomogram for AEG patients based on six clinical associated parameters with data from the Surveillance, Epidemiology, and End Results (SEER) database and an eastern database. Liu et al. [12] proposed a nomogram based on the SEER database for estimating overall survival (OS) in Siewert type II AEG patients who received preoperative radiotherapy. Chen et al. [13] developed and validated a nomogram also based on the SEER database to predict prognosis in patients diagnosed with metastatic Siewert type II AEG. To the best of our understanding, we are the first to attempt to construct a prognostic nomogram for resectable Siewert type II/III AEG based on real-world data, which comprised a total of 396 patients who underwent radical (R0) resection in our center. Moreover, we also aim to identify whether our model provides more accurate prognostic prediction than the current TNM staging system and other prognostic factors.

## Methods

### Patients and study design

We conducted a retrospective analysis of the prospectively collected AEG database of our center. There was a total of 471 cases of Siewert type II/III AEG diagnosed and treated at the Center for Gastrointestinal Surgery, the First Affiliated Hospital, Sun Yat-sen University, from June 2009 to June 2017. Our inclusion criteria were as follow: (1) pathological diagnosis was Siewert type II/III AEG; (2) no history of cancer in other organs; (3) information on the depth of tumor invasion and pathological evaluation records of lymph nodes were complete; (4) patients underwent R0 resection; (5) all clinicopathological and long-term follow-up patient data were complete. Patients who received neoadjuvant chemotherapy were excluded. For those patients with pTNM stage II to IV who could tolerate chemotherapy, adjuvant chemotherapy was routinely recommended, which consisted of either single-agent 5-fluorouracil (5-FU) or a combination of 5-FU and cisplatin/oxaliplatin. Finally, 396 cases were included in this study. They were then divided into three cohorts. 203 cases of them from June 2009 to December 2013 were enrolled into training cohort for establishing a prognostic nomogram; 88 cases of them from January 2014 to June 2015 were enrolled into validation-1 cohort to verify the predictive accuracy of the nomogram in 2-, 3- and 5-year survival; and all remaining 105 cases from July 2015 to June 2017 were enrolled into validation-2 cohort to validate the predictive accuracy of the nomogram in 2- and 3-year survival. OS was defined as the time in months from the operation to the date of AEG-related death or the date of the last follow-up. We defined disease-free survival (DFS) as the time in months from operation until developing a recurrence. Pathological diagnosis was certified by two independent pathologists. The clinical and pathological characteristics and follow-up data of these patients were collected and stored in the database of our center by a team of research assistants. This study strictly complied with the Transparent Reporting of a multivariate prediction model for Individual Prediction or Diagnosis (TRIPOD) guidelines [14].

### Demographics and clinicopathologic characteristics

The original data of demographics and clinicopathologic features of patients with Siewert type II/III AEG were exported from our hospital’s electronic medical records system. The included demographic and clinicopathological variables were listed in Table 1. Blood transfusion indicated patients received an intraoperative blood transfusion as needed. Organ invasion indicated adjacent organs invaded by AEG tumor. The pathological TNM (pTNM) stage of each patient was restaged based on the 8th AJCC/UICC TNM staging system. Postoperative complications were measured by the Clavien-Dindo classification, and 0 indicated no complications. Three lymph node metastasis variables were included for analysis, namely, the positive lymph node count (PLN), lymph node ratio (LNR), and log odds of positive lymph node (LODDS). The LNR was calculated with PLN count / total number of examined lymph nodes. The LODDS was calculated with log_e_[(PLN count + 0.5) / (negative lymph node count + 0.5)]. In the subsequent construction of nomogram, PLN, LNR and LODDS were all categorized into three levels with the optimal cut-off point identified by X-tile software (version 3.6.1) [15].

**Table 1:** Demographics and clinicopathologic characteristics of patients with Siewert type II/III AEG

Variable	Training cohort (*n* = 203)		Validation-1 cohort (*n* = 88)		Validation-2 cohort (*n* = 105)	
	n	%	n	%	n	%
Gender						
Male	159	78.33	62	70.45	86	81.90
Female	44	21.67	26	29.55	19	18.10
Age (year)						
≤ 40	9	4.43	2	2.27	3	2.86
41–50	15	7.39	8	9.09	5	4.76
51–60	60	29.56	16	18.18	20	19.05
> 60	119	58.62	62	70.45	77	73.33
Family history of cancer						
No	175	86.21	76	86.36	94	89.52
Yes	28	13.79	12	13.64	11	10.48
Blood transfusion						
No	143	70.44	65	73.86	81	77.14
< 500 mL	25	12.32	11	12.50	10	9.52
500-1000 mL	25	12.32	10	11.36	7	6.67
> 1000 mL	10	4.93	2	2.27	**7**	6.67
Siewert type						
II	119	58.62	49	55.68	64	60.95
III	84	41.38	39	44.32	41	39.05
Organ invasion						
Absence	147	72.41	62	70.45	79	75.24
Presence	56	27.59	26	29.55	26	24.76
Serosal infiltration						
S0	49	24.14	22	25.00	16	15.24
S1	57	28.08	28	31.82	38	36.19
S2	69	33.99	24	27.27	39	37.14
S3	28	13.79	14	15.91	12	11.43
Borrmann type						
I	12	5.91	9	10.23	6	5.71
II	35	17.24	30	34.09	25	23.81
III	138	67.98	44	50.00	66	62.86
IV	18	8.87	5	5.68	8	7.62
PRM						
< 3 cm	12	5.91	6	6.82	10	9.52
3-5 cm	148	72.91	72	81.82	87	82.86
> 5 cm	43	21.18	10	11.36	8	7.62
DRM (cm)						
Median	12		15		15	
Range	0–30		0–35		0–30	
pTNM stage						
I	13	6.40	12	13.64	14	13.33
II	46	22.66	19	21.59	20	19.05
III	116	57.14	53	60.23	64	60.95
IV	28	13.79	4	4.55	7	6.67
Grade of differentiation						
Well	4	1.97	2	2.27	0	0.00
Moderate	62	30.54	29	32.95	27	25.71
Poor	137	67.49	57	64.77	78	74.29
Complications						
0-I	166	81.77	74	84.09	78	74.29
II-V	37	18.23	14	15.91	27	25.71
PLN						
Median	3		2		3	
Range	0–54		0–30		0–46	
LNR						
Median	0.09		0.04		0.08	
Range	0–0.98		0–0.63		0–0.80	
LODDS						
Median	−2.20		−2.83		−2.36	
Range	−4.84 – 3.27		−4.98 – 0.48		−5.28 – 1.30	
FOBT						
Negative	173	85.22	75	85.23	80	76.19
Positive	30	14.78	13	14.77	25	23.81
WBC (×10^9^/L)						
Median	6.02		6.02		5.98	
Range	2.37–15.01		3.08–13.97		2.90–11.76	
Neut (×10^9^/L)						
Median	3.25		3.54		3.60	
Range	0.55–14.11		1.25–9.93		0.96–8.39	
Hb (g/L)						
Median	128		126		125	
Range	37–166		46–155		39–158	
HCT						
Median	0.380		0.380		0.377	
Range	0.126–0.481		0.152–0.494		0.136–0.474	
PLT (× 10^9^/L)						
Median	229		229		225	
Range	67–676		53–620		61–617	
Blood type						
A	55	27.09	26	29.55	32	30.48
AB	16	7.88	6	6.82	8	7.62
B	51	25.12	18	20.45	22	20.95
O	81	39.90	38	43.18	43	40.95
ALB (g/L)						
Median	39.4		38.3		38.0	
Range	15.0–50.1		24.8–67.0		20.0–49.7	
GLB (g/L)						
Median	25.9		26.3		27.0	
Range	10.0–47.0		17.6–40.0		15.8–40.4	
AFP (ug/L)						
Median	3.41		2.59		2.80	
Range	0–1217.83		0–219.25		0–5968.13	
CEA (ug/L)						
Median	2.94		2.33		3.27	
Range	0–465.21		0–137.51		0–314.68	
CA-125 (U/mL)						
Median	9.70		8.20		10.40	
Range	0–251.10		0–35.80		0–118.70	
CA 19–9 (U/mL)						
Median	9.39		11.68		9.22	
Range	0–11,134.24		0–7941.43		0–12,000	
Surgical resection						
Total stomach	49	24.14	19	21.59	24	22.86
Total stomach + esophagus ± other organs	135	66.50	61	69.32	71	67.62
Total stomach + other organs	19	9.36	8	9.09	10	9.52

*Abbreviations*: *AFP* α-fetoprotein, *ALB* albumin, *CA125* cancer antigen 125, *CA 19–9* cancer antigen 19–9, *CEA* carcino-embryonic antigen, *DRM* distal resection margin, *FOBT* fecal occult blood test, *GLB* globulin, *Hb* hemoglobin, *HCT* hematocrit, *LNR* lymph node ratio, *LODDS* log odds of positive lymph node, *Neut* neutrophil, *PLN* positive lymph node, *PLT* platelet, *PRM* proximal resection margin, *pTNM* pathological tumor node metastasis, *WBC* white blood cell

### Follow-up

All the patients enrolled underwent postoperative follow-up once every 3 months in the first 2 years, then every 6 months in the following 3 years and thereafter annually. Telephone or e-mail follow-up interviews were administered at random. During each follow-up visit, detailed history taking and physical examination were conducted by experienced doctors. Blood was sampled for serum α-fetoprotein (AFP), carcino-embryonic antigen (CEA), cancer antigen 125 (CA125) and CA 19–9. Abdominal CT (once every 6 months) and electronic gastroscopy (annually) were performed, or conducted at a shorter interval when carcinoma recurrence or metastasis was suspected. Further detailed investigations would be performed if clinically indicated. The study was censored on July 31, 2020.

### Statistical analysis

Categorical variables were grouped according to the classification scheme that clinical doctors interested in, and decisions on the grouping were made before subsequent analyses. Data of distal resection margin (DRM) and blood test results, namely, white blood cell count (WBC), neutrophil count (Neut), hemoglobin (Hb), hematocrit (HCT), platelet count (PLT), albumin (ALB), globulin (GLB), AFP, CEA, CA-125, and CA 19–9 were processed into categorical variables based on their median value of training cohort. The Cox proportional hazard regression model was adopted to identify factors that were independently correlated with OS and DFS. Univariate and multivariate analyses were performed in Statistic Package for Social Science (SPSS) 18.0 software. Only a factor that was *P* < 0.05 in univariate analysis could be further adopted for multivariate analysis with ‘Forward LR’ methods. The nomogram was constructed based on multivariate Cox regression coefficients by adopting the ‘rms’ package in R software (version 4.0.3). The performance of the nomogram was measured using Harrell’s concordance index (C-index) with the ‘survival’ package. Receiver operating characteristic (ROC) curve analysis was carried out with the ‘survivalROC’ package to evaluate the prognostic predictive accuracy of the nomogram. Calibration plots were generated to compare the consistency of predicted and observed survival probabilities. With SPSS 18.0, Kaplan-Meier survival curves were constructed for included prognostic factors of the nomogram and the difference of these groups was calculated by log-rank test. A two-tailed *P* < 0.05 was considered statistically significant.

## Results

### Demographics and clinicopathologic features of patients

**Fig. 1:**
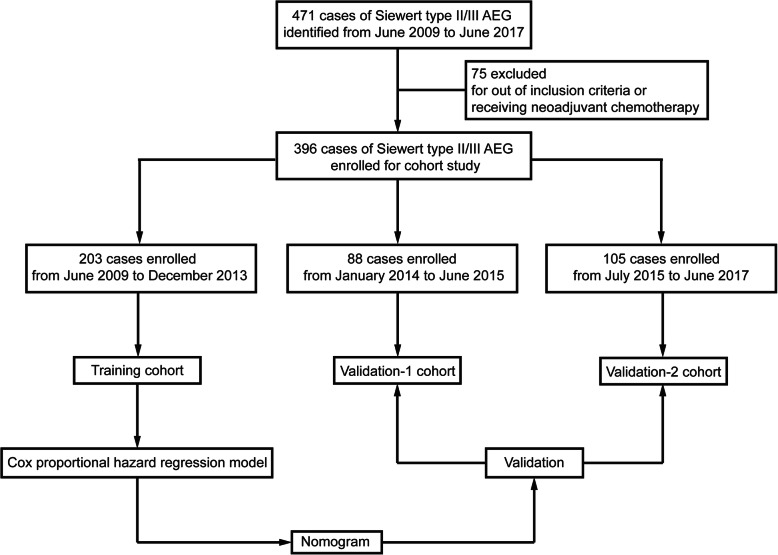
Flow diagram showing steps involved in construction and validation of a nomogram

The design of this study was briefly illustrated in Fig. 1. A total of 396 patients diagnosed as Siewert type II/III AEG were comprised in this current study. Then they were divided into three cohorts: training cohort (*n* = 203), validation-1 cohort (*n* = 88) and validation-2 cohort (*n* = 105). The demographics and clinicopathologic characteristics of these patients are listed in Table 1. There was a total of 29 variables included for analysis, all of which were real-world data exported from the electronic medical records system of our hospital.

### OS and DFS of the three cohorts

The median follow-up time was 99 months in the training cohort, 70 months in the validation-1 cohort, and 47 months in the validation-2 cohort. The median OS was 47 months in the training cohort, and the postoperative 1-, 3-, and 5-year OS was 83.7, 56.7, and 46.6%. The median DFS was 39 months in the training cohort, and the postoperative 1-, 3-, and 5-year DFS was 79.3, 52.4, and 43.9%. In the validation-1 cohort, the postoperative 1-, 3-, and 5-year OS was 87.4, 66.4, and 60.2%, and the postoperative 1-, 3-, and 5-year DFS was 80.6, 61.8, and 58.2%. In the validation-2 cohort, the postoperative 1- and 3-year OS was 86.7 and 58.9%, and the postoperative 1- and 3-year DFS was 84.8 and 54.1%.

### Prognostic factors in the three cohorts

The results of the univariate analysis of factors associated with OS are shown in Table 2. We found in the training cohort, blood transfusion, organ invasion, serosal infiltration, Borrmann type, pTNM stage, grade of differentiation, PLN, LNR, LODDS, PLT, ALB, and CA-125 were correlated with OS; in the validation-1 cohort, organ invasion, serosal infiltration, Borrmann type, DRM, grade of differentiation, complications, PLN, LNR, and LODDS were correlated with OS; and in the validation-2 cohort, blood transfusion, organ invasion, serosal infiltration, Borrmann type, pTNM stage, grade of differentiation, PLN, LNR, LODDS, and fecal occult blood test (FOBT) were correlated with OS.

**Table 2:** Univariate analysis of factors associated with overall survival in patients with Siewert type II/III AEG

Variable	Training cohort (*n* = 203)		Validation-1 cohort (*n* = 88)		Validation-2 cohort (*n* = 105)	
	HR (95%CI)	*P*	HR (95%CI)	*P*	HR (95%CI)	*P*
Gender (vs. Male)	0.926 (0.596–1.436)	0.730	1.623 (0.812–3.243)	0.170	1.359 (0.675–2.734)	0.390
Age (vs. ≤40)	0.819 (0.659–1.018)	0.073	0.981 (0.629–1.527)	0.931	1.079 (0.716–1.627)	0.717
Family history of cancer (vs. No)	0.658 (0.370–1.170)	0.154	0.775 (0.433–1.387)	0.391	1.172 (0.739–1.857)	0.500
Blood transfusion (vs. No)	1.300 (1.091–1.548)	**0.003**	1.194 (0.821–1.738)	0.353	1.471 (1.110–1.949)	**0.007**
Siewert type (vs. II)	1.010 (0.706–1.446)	0.956	1.262 (0.644–2.471)	0.498	1.116 (0.623–1.999)	0.711
Organ invasion (vs. Absence)	2.402 (1.653–3.492)	**< 0.001**	2.189 (1.110–4.315)	**0.024**	2.510 (1.387–4.543)	**0.002**
Serosal infiltration (vs. S0)	1.556 (1.292–1.875)	**< 0.001**	1.541 (1.104–2.150)	**0.011**	2.030 (1.432–2.876)	**< 0.001**
Borrmann type (vs. I)	1.670 (1.223–2.280)	**0.001**	1.716 (1.034–2.849)	**0.037**	1.887 (1.152–3.093)	**0.012**
PRM (vs. < 3 cm)	0.862 (0.601–1.237)	0.421	0.914 (0.387–2.157)	0.914	1.048 (0.517–2.126)	0.896
DRM (vs. < 12 cm)	0.805 (0.565–1.147)	0.229	0.479 (0.244–0.941)	**0.033**	0.901 (0.488–1.664)	0.739
pTNM stage (vs. I-II)	3.128 (1.913–5.112)	**< 0.001**	2.121 (0.959–4.690)	0.063	2.554 (1.233–5.290)	**0.012**
Grade of differentiation (vs. Well)	1.154 (1.074–2.248)	**0.019**	3.140 (1.331–7.409)	**0.009**	2.634 (1.178–5.889)	**0.018**
Complications (vs. 0-I)	1.245 (0.791–1.960)	0.343	3.083 (1.467–6.479)	**0.003**	1.442 (0.771–2.699)	0.252
PLN^a^	1.059 (1.043–1.074)	**< 0.001**	1.067 (1.019–1.117)	**0.005**	1.061 (1.035–1.088)	**< 0.001**
LNR^a^	15.192 (8.172–30.981)	**< 0.001**	29.957 (5.339–168.076)	**< 0.001**	20.054 (5.736–70.106)	**< 0.001**
LODDS^a^	1.548 (1.395–1.719)	**< 0.001**	1.598 (1.261–2.026)	**< 0.001**	1.425 (1.170–1.736)	**< 0.001**
FOBT (vs. Negative)	0.883 (0.535–1.456)	0.626	1.553 (0.676–3.567)	0.300	2.359 (1.296–4.292)	**0.005**
WBC (vs. < 6.02)	1.251 (0.877–1.783)	0.216	0.577 (0.291–1.144)	0.115	0.829 (0.467–1.471)	0.521
Neut (vs. < 3.25)	1.166 (0.818–1.660)	0.396	0.624 (0.318–1.225)	0.171	1.072 (0.591–1.944)	0.819
Hb (vs. < 128)	0.780 (0.547–1.113)	0.171	1.121 (0.572–2.197)	0.738	1.087 (0.609–1.939)	0.778
HCT (vs. < 0.380)	0.797 (0.559–1.137)	0.210	1.298 (0.660–2.555)	0.450	0.795 (0.445–1.418)	0.436
PLT (vs. < 229)	1.492 (1.044–2.131)	**0.028**	0.531 (0.268–1.053)	0.070	0.887 (0.500–1.573)	0.681
Blood type (vs. A)	0.939 (0.813–1.085)	0.396	1.263 (0.952–1.675)	0.106	0.830 (0.669–1.030)	0.091
ALB (vs. < 39.4)	0.674 (0.472–0.964)	**0.031**	0.897 (0.449–1.793)	0.759	1.159 (0.643–2.089)	0.623
GLB (vs. < 25.9)	0.853 (0.598–1.216)	0.379	1.960 (0.937–4.101)	0.074	1.837 (0.983–3.434)	0.057
AFP (vs. < 3.41)	0.790 (0.554–1.126)	0.193	1.358 (0.680–2.712)	0.386	1.415 (0.790–2.535)	0.243
CEA (vs. < 2.94)	1.283 (0.899–1.930)	0.170	0.951 (0.480–1.884)	0.886	1.343 (0.755–2.387)	0.315
CA-125 (vs. < 9.70)	1.561 (1.090–2.236)	**0.015**	1.424 (0.719–2.820)	0.310	1.404 (0.774–2.548)	0.264
CA 19–9 (vs. < 9.39)	1.395 (0.977–1.991)	0.067	0.890 (0.441–1.800)	0.747	1.453 (0.820–2.576)	0.201

*Abbreviations*: *AFP* α-fetoprotein, *ALB* albumin, *CA125* cancer antigen 125, *CA 19–9* cancer antigen 19–9, *CEA* carcino-embryonic antigen, *CI* confidence interval, *DRM* distal resection margin, *FOBT* fecal occult blood test, *GLB* globulin, *Hb* hemoglobin, *HCT* hematocrit, *HR* hazard ratio, *LNR* lymph node ratio, *LODDS* log odds of positive lymph node, *Neut* neutrophil, *PLN* positive lymph node, *PLT* platelet, *PRM* proximal resection margin, *pTNM* pathological tumor node metastasis, *WBC* white blood cell

^a^Continuous variables

The results of the univariate analysis of factors associated with DFS are shown in Table 3. We found in the training cohort, age, blood transfusion, organ invasion, serosal infiltration, Borrmann type, pTNM stage, grade of differentiation, PLN, LNR, LODDS, and PLT were correlated with DFS; in the validation-1 cohort, organ invasion, serosal infiltration, Borrmann type, grade of differentiation, complications, PLN, LNR, LODDS and GLB were correlated with DFS; and in the validation-2 cohort, blood transfusion, organ invasion, serosal infiltration, Borrmann type, pTNM stage, grade of differentiation, PLN, LNR, LODDS, and FOBT were correlated with DFS.

**Table 3:** Univariate analysis of factors associated with disease-free survival in patients with Siewert type II/III AEG

Variable	Training cohort (*n* = 203)		Validation-1 cohort (*n* = 88)		Validation-2 cohort (*n* = 105)	
	HR (95%CI)	*P*	HR (95%CI)	*P*	HR (95%CI)	*P*
Gender (vs. Male)	0.952 (0.618–1.466)	0.823	1.419 (0.718–2.803)	0.314	1.144 (0.574–2.278)	0.702
Age (vs. ≤40)	0.799 (0.643–0.993)	**0.043**	1.002 (0.642–1.563)	0.994	0.936 (0.659–1.330)	0.711
Family history of cancer (vs. No)	0.645 (0.363–1.146)	0.135	0.958 (0.604–1.519)	0.855	1.098 (0.694–1.738)	0.689
Blood transfusion (vs. No)	1.247 (1.046–1.486)	**0.014**	1.124 (0.772–1.637)	0.541	1.361 (1.033–1.793)	**0.028**
Siewert type (vs. II)	1.111 (0.780–1.581)	0.560	1.271 (0.661–2.443)	0.472	0.995 (0.571–1.735)	0.986
Organ invasion (vs. Absence)	2.470 (1.708–3.572)	**< 0.001**	1.955 (1.006–3.800)	**0.048**	2.145 (1.208–3.812)	**0.009**
Serosal infiltration (vs. S0)	1.474 (1.228–1.770)	**< 0.001**	1.434 (1.040–1.979)	**0.028**	1.947 (1.404–2.699)	**< 0.001**
Borrmann type (vs. I)	1.573 (1.160–2.133)	**0.004**	1.640 (1.007–2.671)	**0.047**	1.990 (1.235–3.207)	**0.005**
PRM (vs. < 3 cm)	0.846 (0.595–1.204)	0.353	1.221 (0.515–2.894)	0.650	1.198 (0.623–2.302)	0.589
DRM (vs. < 12 cm)	0.761 (0.536–1.079)	0.126	0.598 (0.311–1.152)	0.124	1.075 (0.591–1.956)	0.812
pTNM stage (vs. I-II)	3.128 (1.934–5.058)	**< 0.001**	1.660 (0.800–3.445)	0.174	2.332 (1.199–4.537)	**0.013**
Grade of differentiation (vs. Well)	1.485 (1.036–2.127)	**0.031**	3.455 (1.468–8.133)	**0.005**	2.676 (1.260–5.685)	**0.010**
Complications (vs. 0-I)	1.220 (0.782–1.904)	0.381	3.504 (1.711–7.175)	**0.001**	1.165 (0.632–2.148)	0.624
PLN^a^	1.052 (1.037–1.067)	**< 0.001**	1.060 (1.014–1.108)	**0.011**	1.059 (1.035–1.084)	**< 0.001**
LNR^a^	11.958 (6.262–22.834)	**< 0.001**	31.083 (5.305–182.122)	**< 0.001**	16.295 (4.944–53.705)	**< 0.001**
LODDS^a^	1.485 (1.343–1.641)	**< 0.001**	1.543 (1.222–1.948)	**< 0.001**	1.414 (1.176–1.700)	**< 0.001**
FOBT (vs. Negative)	0.965 (0.592–1.572)	0.887	1.444 (0.632–3.298)	0.383	1.904 (1.067–3.398)	**0.029**
WBC (vs. < 6.02)	1.180 (0.832–1.675)	0.353	0.653 (0.338–1.260)	0.204	0.961 (0.561–1.648)	0.886
Neut (vs. < 3.25)	1.113 (0.784–1.578)	0.550	0.682 (0.354–1.311)	0.251	1.164 (0.659–2.056)	0.600
Hb (vs. < 128)	0.811 (0.571–1.151)	0.241	1.348 (0.700–2.593)	0.372	1.216 (0.707–2.091)	0.480
HCT (vs. < 0.380)	0.810 (0.571–1.149)	0.238	1.527 (0.787–2.963)	0.211	0.945 (0.551–1.622)	0.838
PLT (vs. < 229)	1.512 (1.063–2.151)	**0.022**	0.524 (0.270–1.018)	0.056	0.995 (0.581–1.705)	0.986
Blood type (vs. A)	0.937 (0.812–1.082)	0.379	1.325 (0.999–1.755)	0.050	0.876 (0.714–1.074)	0.202
ALB (vs. < 39.4)	0.737 (0.518–1.047)	0.089	0.938 (0.479–1.834)	0.851	1.323 (0.766–2.286)	0.315
GLB (vs. < 25.9)	0.798 (0.562–1.134)	0.208	2.177 (1.049–4.517)	**0.037**	1.397 (0.797–2.450)	0.243
AFP (vs. < 3.41)	0.750 (0.528–1.065)	0.108	1.219 (0.617–2.407)	0.569	1.270 (0.729–2.214)	0.399
CEA (vs. < 2.94)	1.301 (0.916–1.849)	0.141	1.119 (0.580–2.161)	0.737	1.348 (0.785–2.317)	0.279
CA-125 (vs. < 9.70)	1.410 (0.991–2.008)	0.056	1.503 (0.775–2.917)	0.228	1.407 (0.802–2.468)	0.234
CA 19–9 (vs. < 9.39)	1.328 (0.935–1.887)	0.113	0.768 (0.384–1.536)	0.456	1.618 (0.943–2.776)	0.081

*Abbreviations*: *AFP* α-fetoprotein, *ALB* albumin, *CA125* cancer antigen 125, *CA 19–9* cancer antigen 19–9, *CEA* carcino-embryonic antigen, *CI* confidence interval, *DRM* distal resection margin, *FOBT* fecal occult blood test, *GLB* globulin, *Hb* hemoglobin, *HCT* hematocrit, *HR* hazard ratio, *LNR* lymph node ratio, *LODDS* log odds of positive lymph node, *Neut* neutrophil, *PLN* positive lymph node, *PLT* platelet, *PRM* proximal resection margin, *pTNM* pathological tumor node metastasis, *WBC* white blood cell

^a^Continuous variables

Moreover, the identified 12 prognostic factors of the training cohort were further analyzed in multivariate analysis. We found in the training cohort, blood transfusion, pTNM stage, and LODDS were independent prognostic factors for OS; in the validation-1 cohort, organ invasion, LODDS and PLT were independent prognostic factors for OS; and in the validation-2 cohort, serosal infiltration and LODDS were independent prognostic factors for OS (Table 4). In addition, the multivariate analysis also demonstrated in the training cohort, organ invasion, and LODDS were independent prognostic factors for DFS; in the validation-1 cohort, LODDS and PLT were independent prognostic factors for DFS; and in the validation-2 cohort, serosal infiltration and PLN were independent prognostic factors for DFS (Table 5).

**Table 4:** Multivariate analysis of factors associated with overall survival in patients with Siewert type II/III AEG

Variable	Training cohort (*n* = 203)		Validation-1 cohort (*n* = 88)		Validation-2 cohort (*n* = 105)	
	HR (95%CI)	*P*	HR (95%CI)	*P*	HR (95%CI)	*P*
Blood transfusion (vs. No)	1.260 (1.046–1.517)	**0.015**	NA		NA	
Organ invasion (vs. Absence)	NA		2.124 (1.051–4.293)	**0.036**	NA	
Serosal infiltration (vs. S0)	NA		NA		1.852 (1.287–2.667)	**0.001**
Borrmann type (vs. I)	NA		NA		NA	
pTNM stage (vs. I-II)	1.438 (1.007–2.053)	**0.046**	NA		NA	
Grade of differentiation (vs. Well)	NA		NA		NA	
PLN^a^	NA		NA		NA	
LNR^a^	NA		NA		NA	
LODDS^a^	1.478 (1.322–1.651)	**< 0.001**	1.561 (1.237–1.970)	**< 0.001**	1.323 (1.079–1.623)	**0.007**
PLT (vs. < 229)	NA		0.400 (0.198–0.809)	**0.011**	NA	
ALB (vs. < 39.4)	NA		NA		NA	
CA-125 (vs. < 9.70)	NA		NA		NA	

*Abbreviations*: *ALB* albumin, *CA125* cancer antigen 125, *CI* confidence interval, *HR* hazard ratio, *LNR* lymph node ratio, *LODDS* log odds of positive lymph node, *NA* not available, *PLN* positive lymph node, *PLT* platelet, *pTNM* pathological tumor node metastasis

^a^Continuous variables

**Table 5:** Multivariate analysis of factors associated with disease-free survival in patients with Siewert type II/III AEG

Variable	Training cohort (*n* = 203)		Validation-1 cohort (*n* = 88)		Validation-2 cohort (*n* = 105)	
	HR (95%CI)	*P*	HR (95%CI)	*P*	HR (95%CI)	*P*
Blood transfusion (vs. No)	NA		NA		NA	
Organ invasion (vs. Absence)	1.612 (1.074–2.422)	**0.021**	NA		NA	
Serosal infiltration (vs. S0)	NA		NA		1.894 (1.348–2.660)	**< 0.001**
Borrmann type (vs. I)	NA		NA		NA	
pTNM stage (vs. I-II)	NA		NA		NA	
Grade of differentiation (vs. Well)	NA		NA		NA	
PLN^a^	NA		NA		1.058 (1.032–1.085)	**< 0.001**
LNR^a^	NA		NA		NA	
LODDS^a^	1.408 (1.265–1.568)	**< 0.001**	1.558 (1.249–1.944)	**< 0.001**	NA	
PLT (vs. < 229)	NA		0.458 (0.236–0.889)	**0.021**	NA	
ALB (vs. < 39.4)	NA		NA		NA	
CA-125 (vs. < 9.70)	NA		NA		NA	

*Abbreviations*: *ALB* albumin, *CA125* cancer antigen 125, *CI* confidence interval, *HR* hazard ratio, *LNR* lymph node ratio, *LODDS* log odds of positive lymph node, *NA* not available, *PLN* positive lymph node, *PLT* platelet, *pTNM* pathological tumor node metastasis

^a^Continuous variables

### Nomogram construction for predicting OS of patients with Siewert type II/III AEG

**Fig. 2:**
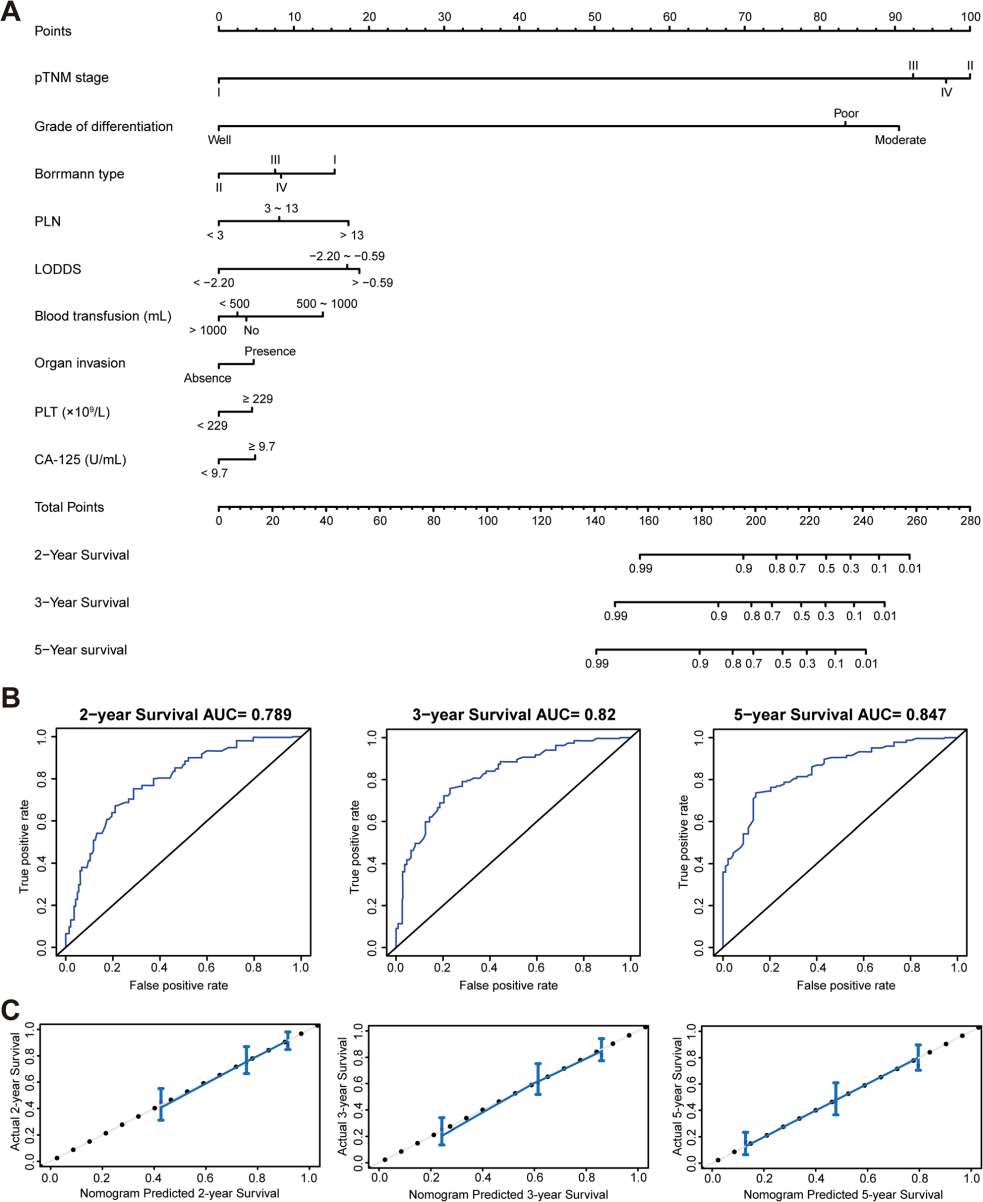
Nomogram construction for predicting OS of patients with Siewert type II/III AEG. **a** The established nomogram based on nine variables for predicting 2-, 3- and 5-year survival. **b** The time-dependent ROC analysis showed the AUC of this nomogram was 0.789 at 2-year, 0.82 at 3-year, and 0.847 at 5-year. **c** Calibration plots showed good agreement for the survival probability at 2-, 3- and 5-year between the nomogram prediction and actual observed results

We attempted to construct a nomogram to develop a quantitative method for predicting the survival probability of patients with Siewert type II/III AEG. As the multivariate analysis in the training cohort showed only three variables were independent prognostic factors for OS, namely blood transfusion, pTNM stage, and LODDS, and the C-index of the nomogram if built based on these three factors was only 0.72 (95% CI, 0.68 to 0.76), we included other prognostic factors identified by univariate analysis into the nomogram to obtain the best model with the highest C-index. The candidate risk factors were organ invasion, serosal infiltration, Borrmann type, grade of differentiation, PLN, LNR, PLT, and CA-125. Moreover, for the convenience of clinical application, continuous variables, namely PLN, LNR, and LODDS, were all categorized into three levels according to the cut-off value determined by X-tile software. In addition, to make the model easy to use and promote, the number of included variables should be limited. Since organ invasion, Borrmann type, grade of differentiation, PLT, and CA-125 were variables that showed information of different aspects, and were complementary to the three independent prognostic factors, they were included into the model. PLN and LNR were correlated with LODDS, and thus we doubted whether including one of them or both would increase the predictive accuracy of this model. We found that if the nomogram was constructed without PLN and LNR, the C-index was 0.75 (95% CI, 0.71 to 0.79), which was similar to that built with LNR added (C-index = 0.75 [95% CI, 0.71 to 0.79]), but lower than that built with PLN added (C-index = 0.76 [95% CI, 0.72 to 0.80]) and that built with both PLN and LNR added (C-index = 0.76 [95% CI, 0.71 to 0.80]). Therefore, we included PLN and excluded LNR. Besides, the information reflected by serosal infiltration was similar to that of the pT stage of the pTNM staging system. By calculation, we found if the nomogram included serosal infiltration, the C-index was 0.76 (95% CI, 0.71 to 0.80), which was similar to the nomogram that built without adding serosal infiltration (C-index = 0.76 [95% CI, 0.72 to 0.80]); and if the model included serosal infiltration and excluded PLN, the C-index was 0.75 (95% CI, 0.71 to 0.79). Consequently, serosal infiltration was also excluded. Finally, a nomogram based on nine variables was established (Fig. 2a). The C-index of each supposed model with different variables included were also shown in Supplementary Table 1.

We then conducted a time-dependent ROC analysis to further evaluate the prognostic prediction value of the nomogram. The results showed the area under the ROC curve (AUC) of this nomogram was 0.789 at 2-year, 0.82 at 3-year, and 0.847 at 5-year (Fig. 2b), which indicated that it was of satisfactory predictive value, especially in predicting 5-year OS. We also drew calibration plots for the probability of survival at 2-, 3- and 5-year after surgery, and results showed good agreement between the nomogram prediction and actual observed results (Fig. 2c).

### Validation of the nomogram for predicting OS of patients with Siewert type II/III AEG

**Fig. 3:**
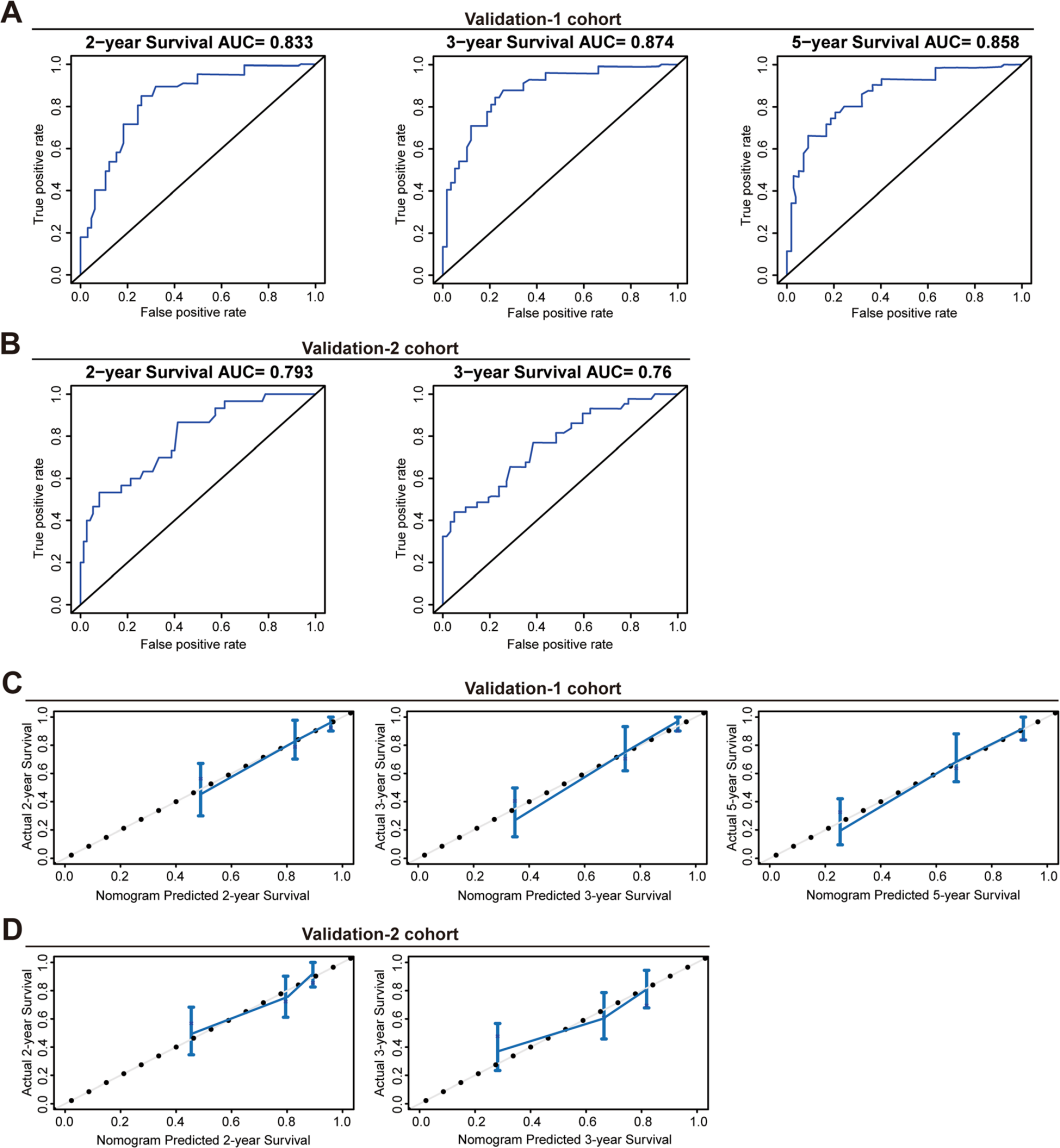
Validation of the nomogram for predicting OS of patients with Siewert type II/III AEG. **a** The time-dependent ROC analysis showed the AUC of this nomogram in the validation-1 cohort was 0.833 at 2-year, 0.874 at 3-year, and 0.858 at 5-year. **b** The time-dependent ROC analysis showed AUC of this nomogram in the validation-2 cohort was 0.793 at 2-year and 0.76 at 3-year. **c** Calibration plots showed the predicted survival probability at 2-, 3- and 5-year was well consistent with that observed in the validation-1 cohort. **d** Calibration plots showed the predicted probability of survival at 2- and 3-year was well consistent with that observed in the validation-2 cohort

We further validated the predictive value of the nomogram in the two validation cohorts. The C-index for OS prediction was 0.79 (95% CI, 0.72 to 0.86) in the validation-1 cohort, and 0.73 (95% CI, 0.67 to 0.80) in the validation-2 cohort. In the validation-1 cohort, time-dependent ROC analysis demonstrated the AUC of this nomogram was 0.833 at 2-year, 0.874 at 3-year, and 0.858 at 5-year (Fig. 3a). In the validation-2 cohort, the AUC of this nomogram was 0.793 at 2-year and 0.76 at 3-year (Fig. 3b). Additionally, in the validation-1 cohort, calibration curve showed good consistency between the predicted and observed survival probability at 2-, 3- and 5-year (Fig. 3c); and in the validation-2 cohort, calibration plots also showed the predicted survival probability at 2- and 3-year was well consistent with that observed (Fig. 3d).

### Comparison of predictive accuracy for OS between nomogram and independent prognostic factors

We also compared the predictive accuracy for OS between nomogram and independent prognostic factors. In the training cohort, blood transfusion, pTNM stage and LODDS were independent prognostic factors for OS, and the C-index of each was respectively 0.51 (95% CI, 0.47 to 0.55), 0.64 (95% CI, 0.60 to 0.69) and 0.69 (95% CI, 0.65 to 0.73), all of which were lower than that of the nomogram (0.76). In the validation-1 cohort, organ invasion, LODDS and PLT were independent prognostic factors, and their C-index were respectively 0.54 (95% CI, 0.46 to 0.61), 0.64 (95% CI, 0.56 to 0.72) and 0.57 (95% CI, 0.49 to 0.66), all of which were lower than that of the nomogram (0.79). In the validation-2 cohort, serosal infiltration and LODDS were independent prognostic factors, and their C-index were respectively 0.67 (95% CI, 0.59 to 0.74) and 0.63 (95% CI, 0.55 to 0.71), all of which were lower than that of the nomogram (0.73). Besides, the C-index of the pTNM stage in the validation-1 and validation-2 cohorts were respectively 0.59 (95% CI, 0.51 to 0.67) and 0.64 (95% CI, 0.57 to 0.70), both of which were lower than that of the nomogram. These results indicated that the nomogram was a helpful predictor for the survival of patients with Siewert type II/III AEG.

### Kaplan-Meier survival curves of the included nine prognostic factors of the nomogram

**Fig. 4:**
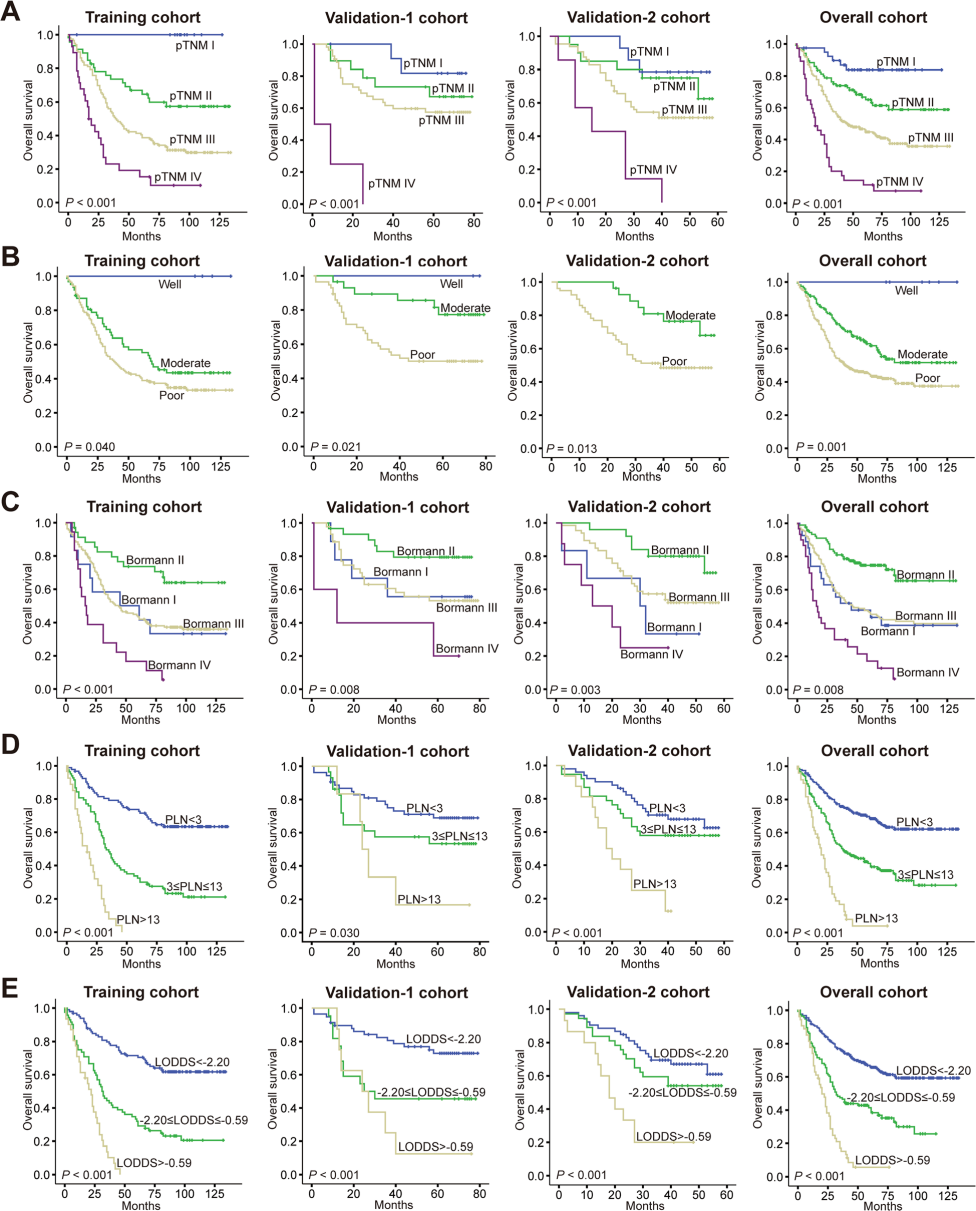
Kaplan-Meier survival curves of five prognostic factors in the training, validation-1, validation-2, and overall cohorts. **a** pTNM stage, (**b**) grade of differentiation, (**c**) Borrmann type, (**d**) PLN, (**e**) LODDS. *P* value was shown respectively in each panel

**Fig. 5:**
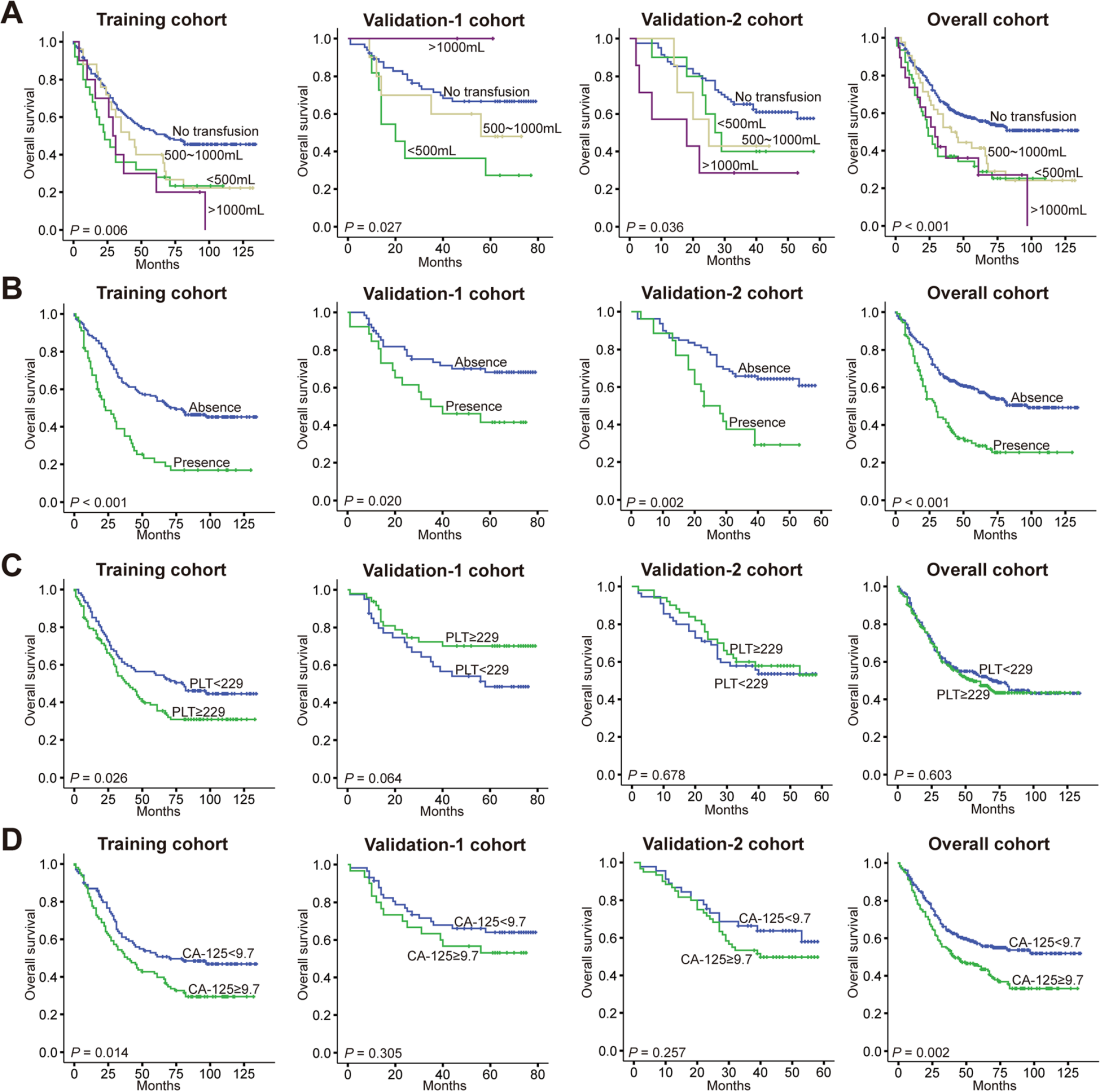
Kaplan-Meier survival curves of four prognostic factors in the training, validation-1, validation-2, and overall cohorts. **a** blood transfusion, (**b**) organ invasion, (**c**) PLT, (**d**) CA-125. *P* value was shown respectively in each panel

Last, we merged data of training cohort, validation-1 cohort and validation-2 cohort into an overall cohort, and then drew Kaplan-Meier survival curves of the included nine prognostic factors of the nomogram, namely, pTNM stage (Fig. 4a), grade of differentiation (Fig. 4b), Borrmann type (Fig. 4c), PLN (Fig. 4d), LODDS (Fig. 4e), blood transfusion (Fig. 5a), organ invasion (Fig. 5b), PLT (Fig. 5c) and CA-125 (Fig. 5d). We found the pTNM stage, grade of differentiation, PLN, LODDS and organ invasion were significant prognostic factors with good discriminative ability. For Borrmann type, patients of Borrmann I and III have similar OS, and patients of Borrmann II have the highest OS, while patients of Borrmann IV have the lowest OS. For blood transfusion, OS of patients received a transfusion of different volume were similar, while the OS of patients without transfusion was significantly higher than others. For PLT, though analysis in the training cohort showed it was a prognostic factor, data shown in two validation cohorts and the overall cohort demonstrated preoperative PLT of patients was not correlated with their OS. For CA-125, though survival curves of two validation cohorts both showed no significant difference between patients of different serum CA-125 levels, data shown in the training cohort and overall cohort demonstrated preoperative serum CA-125 of patients was correlated with their OS.

## Discussion

The clinical staging systems for AEG are still controversial. Recommendations for the staging of AEG in terms of esophageal or gastric schemes have their limitations, since AEG has its specific molecular biology and pathological features. Moreover, there have been no staging systems specifically proposed for postoperative prognostication, and the prediction of the conventional staging systems is of low accuracy. For example, in our study, we observed the C-index of the 8th pTNM stage system in survival prediction was only 0.64 in the training cohort, and in the validation-1 and validation-2 cohorts was respectively 0.59 and 0.64. Nomograms have been proven more accurate in prognostication than the traditional staging systems in several cancers, including AEG [9–12]. But these established nomograms for AEG were mainly based on several variables originated from the SEER database [11–13], while the number of registered variables in the SEER database was limited, and thus the predictive accuracy could not be highly satisfied. We are the first to construct a prognostic nomogram for resectable Siewert type II/III AEG based on real-world data, which contained 29 variables exported from the electronic medical records system of our hospital. Real-world data is invaluable to acquire complementary yet indispensable evidence for preclinical and clinical studies [16, 17]. Unlike nomograms built based on gene expression level, which need gene detection (expensive and hard to be widely applied in clinical setting), nomograms constructed for predicting survival based on real-world data are also valuable for their easiness to use and promote. We at first screened out 12 significant prognostic indicators from the included 29 variables by performing univariate analysis in the training cohort. Next with multivariate analysis, we identified three independent prognostic factors in the training cohort. To obtain an optimal model, we included other prognostic factors identified by univariate analysis into the nomogram. Eventually, a nomogram was established based on nine variables, namely pTNM stage, grade of differentiation, Borrmann type, PLN, LODDS, blood transfusion, organ invasion, PLT, and CA-125, whose C-index in training, validation-1 and validation-2 cohort was respectively 0.76 (95% CI, 0.72 to 0.80), 0.79 (95% CI, 0.72 to 0.86) and 0.73 (95% CI, 0.67 to 0.80), indicating high predictive accuracy. We further proved the superiority of the nomogram in predictive accuracy for OS to pTNM staging system and other independent prognostic factors. At last, we merged the data of the three cohorts into an overall cohort and then performed Kaplan-Meier survival curves of the included nine prognostic factors of the nomogram and verified pTNM stage, grade of differentiation, PLN, LODDS, and organ invasion were prognostic factors with good discriminative ability.

Nomogram is a quantitative model for predicting prognosis of patients and could assist us in implementing clinical decision-making. We could estimate the 2-, 3-, and 5-year survival rate for each Siewert type II/III AEG patient by drafting a vertical line between the total point axis and each of the three prognosis axes. If a patient was estimated to be of high survival rate by the nomogram, gastroenterology practitioners could implement more aggressive postoperative interventions, such as aggressive adjuvant chemotherapy regimens, and surgical resection may again be conducted if relapsed after several years. Conversely, if a patient was estimated to be of low survival rate by the nomogram, the postoperative therapeutic strategy should focus on improving quality of life and prolonging the patient’s survival time, not radical removal of remaining tumor cells.

The only proven significant independent prognostic factor in the three cohorts was LODDS. LODDS is a newly identified promising index for prognosis prediction, which has been verified in several malignancies, such as small bowel adenocarcinoma [18, 19], gastric cancer [20], and AEG [12, 21]. With data from the SEER database, Xu et al. [21] demonstrated LODDS exhibited greater prognostic prediction accuracy in postoperative patients with Siewert type II AEG when compared to using PLN and LNR. Additionally, LODDS could also assist in evaluating survival heterogeneity for patients without positive lymph nodes identified. Also based on the SEER database, Liu et al. [12] proved LODDS was an independent prognostic indicator for patients with Siewert type II AEG after preoperative radiotherapy. However, a majority of patients enrolled in the SEER database were from Western populations, and thus it was ambiguous on the accuracy of LODDS used to predict AEG patients’ prognosis of Eastern populations. Our findings were based on a single center in China and we identified LODDS as an independent prognostic indicator for Siewert type II/III AEG patients. We hope that this study may amplify the clinical application of LODDS.

In this current study, with univariate analysis, we identified several interesting possible prognostic factors for Siewert type II/III AEG patients, namely intraoperative blood transfusion, preoperative PLT, and ALB. We found intraoperative blood transfusion was a detrimental prognostic factor for OS and DFS of Siewert type II/III AEG patients, while the volume of transfusion would not affect their survival. Several clinical studies have reported the unfavorable effects of perioperative blood transfusions on patients’ outcomes after tumor resections [22, 23]. The underlying mechanism was most probably attributed to transfusion-related immunomodulation (TRIM). TRIM is a biological phenomenon that encompasses an activation of immunomodulatory mechanisms caused by blood transfusion. This may lead to immunosuppression, availing cancer recurrence, and metastasis. The TRIM phenomenon might be caused by the following factors: the transfused immunosuppressive cytokines originated from donor components or generated during blood processing, the presence of apoptotic cells and residual leukocytes, the transfer of microparticles and extracellular vesicles loaded with metabolically active growth factor, and the presence of free hemoglobin or bound hemoglobin with extracellular vesicle [24]. Therefore, more restrictive transfusion practices should be advocated for surgical patients. Secondly, we found increased preoperative level of PLT was associated with reduced survival in the training cohort. High PLT is an independent risk factor for cancer-associated venous thromboembolism, which constitutes the second leading cause of mortality in malignant patients [25]. Moreover, recent preclinical and clinical evidence uncover complex crosstalk between cancer cells and platelets [25, 26]. Cancer could directly induce tumor-platelet aggregates, trigger the release of platelet granule and extracellular vesicle, alter the phenotype and RNA profiles of platelet, and stimulate thrombopoiesis. Reciprocally, platelets acquire tumor cell proliferation and growth-enhancing traits via promoting signal pathways associated with proliferation, anti-apoptotic effect, and secretion of prosurvival angiogenic factors. Platelet-tumor cell contact has been shown to increase the survival of metastatic seeds by enhancing tumor cell epithelial-mesenchymal transition. Tumor cell gains the ability to invade epithelial and/or basal membranes, intravasate the blood or lymphatic circulation, and finally extravasate to distant organs. Withal, there has been evidence that platelets may play a role in tumor-cell immune evasion. Hence, this might explain the negative clinical outcomes observed in Siewert type II/III AEG patients with high PLT. Last, we observed favorable survival in patients with high ALB. It has been reported in many studies that serum ALB levels were significantly correlated with the survival of cancer patients [27, 28]. Serum ALB is an indicator of nutritional status. It is widely accepted that an overall nutritional status correlates with the survival of cancer patients [29]. The presence of cancer is detrimental due to its chronic energy-consumptive nature and often results in malnutrition. Thus, that is why patients with low ALB showed poor prognosis in our study. All in all, these three prognostic factors might uncover novel mechanisms underlying AEG relapse and progression.

However, there exist some limitations in this study. First, the prognostic score calculated with the nomogram largely depends on the pTNM stage and grade of differentiation, while the other seven variables seem to play a minor role in score calculation. By further analysis, we found if the nomogram was built with only the two factors, pTNM stage and grade of differentiation, the C-index in the training cohort was 0.65 (95% CI, 0.60 to 0.70), which was markedly lower than that of the current nomogram. Therefore, we think the other seven variables were complementary yet indispensable. Second, neoadjuvant treatment for AEG has currently been an increasing trend of care worldwide except for Stage I. In this view, the pTNM staging system after surgical resection would not work well due to the effect of the precedent treatment, so would this nomogram. Nevertheless, the optimal neoadjuvant treatment strategy remains in question. In a retrospective propensity score-matched analysis of patients with stage II and III AEG from 10 European centers, compared to the neoadjuvant chemotherapy group, pathologically complete remissions and R0 resections were more frequent in the neoadjuvant radiochemotherapy group at the cost of increased postoperative anastomotic leak and somewhat increased 90-day postoperative mortality [30]. Therefore, a clear preference for either treatment is not yet available [31, 32]. Additionally, the optimal neoadjuvant therapeutic cycle is yet to be established. If neoadjuvant treatment takes too long, the patient may miss the best time for surgery. Subsequently, neoadjuvant treatment has not been widely accepted by AEG patients, especially those stage II-III patients. Thus, our nomogram could still be used for those patients who refused neoadjuvant treatment. In future, we will attempt to construct a nomogram specifically for patients receiving neoadjuvant treatment. Another major limitation of this study was that the nomogram was constructed based on data from a single center in China. It needs to be further verified in external validation cohorts of other institutions, ideally in different regions. Yet, different from nomograms built based on a public database, such as the SEER database, this nomogram was established based on real-world data, including blood test results, most of which could not be found in the public databases. Thus, it is hard to be validated by using data from a public database. We are trying to cooperate with other medical centers to establish a more validated nomogram in future studies, and we would appreciate it if any research team could provide the invaluable real-world data of AEG patients treated in their center. Last but not least, due to the lack of widely accepted predictive models, we as well as other survival prediction model developers, could only compare the developed prediction model to the TNM classification, which has been recognized as an important reference basis for clinical decision-making. However, the TNM classification has not been developed as a survival prediction model for an individual patient. Therefore, we hope a widely accepted prediction model will be established for AEG patients someday, and thus the comparison can be more sensible. In the future, with the application of high-throughput sequencing in clinic, the predictive accuracy might be further improved if the nomogram enrolled some genomic characteristics, such as microsatellite instability (MSI), chromosomal instability (CIN), Epstein-Barr virus-associated (EBV), and genomically stable (GS) [33, 34]. All in all, despite these limitations, we believe this nomogram could indeed help gastroenterology practitioners to implement clinical decision-making for Siewert type II/III AEG patients.

## Conclusions

In summary, the current study identified several novel prognostic factors and constructed a prognostic nomogram that is better in survival prediction than the pTNM staging system and other independent prognostic factors for Siewert type II/III AEG patients after surgery. This model could assist gastroenterology practitioners to estimate survival more precisely and thus implement evidence-based decision-making.

## Supplementary Information

**Additional file 1: Supplementary Table 1.** The C-index of each supposed model for overall survival prediction with different variables included.

## Data Availability

The data that support the findings of this study are available from the corresponding author upon reasonable request.

## Abbreviations

AEG: Adenocarcinoma of the esophagogastric junction
EGJ: Esophagogastric junction
UICC: Union for International Cancer Control
AJCC: American Joint Committee on Cancer
TNM: Tumor-node-metastasis
SEER: Surveillance, Epidemiology, and End Results
OS: Overall survival
DFS: Disease-free survival
TRIPOD: Transparent Reporting of a multivariate prediction model for Individual Prediction or Diagnosis
pTNM: Pathological TNM
PLN: Positive lymph node
LNR: Lymph node ratio
LODDS: Log odds of positive lymph node
AFP: α-fetoprotein
CEA: Carcino-embryonic antigen
CA125: Cancer antigen 125
CA19–9: Cancer antigen 19–9
WBC: White blood cell
Neut: Neutrophil
Hb: Hemoglobin
HCT: Hematocrit
PLT: Platelet
ALB: Albumin
GLB: Globulin
FOBT: Fecal occult blood test
PRM: Proximal resection margin
DRM: Distal resection margin
C-index: Harrell’s concordance index
ROC: Receiver operating characteristic
AUC: Area under the ROC curve
MSI: Microsatellite instability
CIN: Chromosomal instability
EBV: Epstein-Barr virus-associated
GS: Genomically stable
