# Supplementary material for: Development and validation of a survival nomogram for patients with Siewert type II/III adenocarcinoma of the esophagogastric junction based on real-world data
Source: BMC Cancer. 2021 May 10;21:532. doi: 10.1186/s12885-021-08249-x (PMC8111941; doi:10.1186/s12885-021-08249-x)
Supplement: Supplementary file 1 — Additional file 1: Supplementary Table 1. The C-index of each supposed model for overall survival prediction with different variables included. [file 12885_2021_8249_MOESM1_ESM.docx]

**Supplementary Table 1** The C-index of each supposed model for overall survival prediction with different variables included

| Variables included | C-index (95% CI) |
| --- | --- |
| blood transfusion, pTNM stage, LODDS | 0.72 (0.68 to 0.76) |
| blood transfusion, pTNM stage, LODDS, Borrmann type, grade of differentiation, organ invasion, PLT, CA-125 | 0.75 (0.71 to 0.79) |
| blood transfusion, pTNM stage, LODDS, Borrmann type, grade of differentiation, organ invasion, PLT, CA-125, LNR | 0.75 (0.71 to 0.79) |
| blood transfusion, pTNM stage, LODDS, Borrmann type, grade of differentiation, organ invasion, PLT, CA-125, PLN | **0.76 (0.72 to 0.80)** |
| blood transfusion, pTNM stage, LODDS, Borrmann type, grade of differentiation, organ invasion, PLT, CA-125, PLN, LNR | 0.76 (0.71 to 0.80) |
| blood transfusion, pTNM stage, LODDS, Borrmann type, grade of differentiation, organ invasion, PLT, CA-125, PLN, serosal infiltration | 0.76 (0.71 to 0.80) |
| blood transfusion, pTNM stage, LODDS, Borrmann type, grade of differentiation, organ invasion, PLT, CA-125, serosal infiltration | 0.75 (0.71 to 0.79) |

**Abbreviations:** CA125, cancer antigen 125; CI, confidence interval; LNR, lymph node ratio; LODDS, log odds of positive lymph node; PLN, positive lymph node; PLT, platelet; pTNM, pathological tumor node metastasis.
